# Variance in multiplex suspension array assays: A distribution generation machine for multiplex counts

**DOI:** 10.1186/1742-4682-5-3

**Published:** 2008-01-28

**Authors:** Brian P Hanley

**Affiliations:** 1 Microbiology Graduate Group, University of California, Davis, CA 95616, USA; 2 BW Education and Forensics, 2710 Thomes Avenue, Cheyenne, Wyoming 82001, USA

## Abstract

**Background:**

This study attempted to replicate Luminex experimental results for large numbers of beads per classifier using multiplexed assays and routine instrument use conditions.

**Conclusion:**

Using larger numbers of microspheres per classifier highlights a fundamental stochastic distribution of bead counts issue complicated by other factors. The more classifiers and the higher the count required per classifier there are, the more apparent the distribution of counts per classifier will be, and the more microspheres are required. Additional problems have been identified. Alternate methods of improving precision and reliability are recommended such as intraplexing and multi-well sample replicates to improve precision and confidence.

## Background

In a study by Jacobson et al. [1] up to 1000 microspheres were acquired for a single classifier. Those results showed improved confidence intervals and more reliable mean values for 1000 microspheres. The current study attempted to replicate those experimental results using multiplex assays. A multiplex assay is one where more than one classifier set of microspheres, each classifier set bearing an assay, are combined together in a mix and inserted into a single sample. Multiplexing is intended to conduct multiple assays simultaneously from a very limited sample.

In order to understand the problem better, an illustrative metaphor will be used of a swimming pool filled with M&Ms (3 mm candy coated pieces of chocolate, from Mars, Inc. USA) of different colors. In this example, the swimming pool is analogous to a single sample well, and one M&M is analogous to a single microsphere. The populations of M&Ms that are of the same color are analogous to a classifier set.

An equal number of M&Ms in each of 100 colors are put into the swimming pool, and it is assumed the M&Ms are fully mixed having no artifacts such as differential density of one color M&M leading to concentration at the bottom or top. A large barrel of M&Ms is randomly scooped from the pool, and a scoop of M&Ms is removed from the barrel. Finally, all M&Ms in the scoop are thrown high into the air, and those that land within an arbitrary 6-foot diameter circle are counted for each color. This 6 foot diameter circle corresponds to what is read by a flow cytometer that is able to classify by color.

What will be understood from the above thought experiment is that there will not be an equal number of each color of M&Ms in the 6-foot circle. If one collects the counts obtained for each M&M color, then categorizes them into a histogram with 5 to 20 different bins (categories on regular intervals), plotting range of M&M counts in each histogram bin on the X axis against number of times a count is found within that X axis bin range, what one expects to see is a distribution of counts.

This M&M metaphor is analogous to how microspheres are presented to the flow cell of a Luminex flow cytometer: For the Luminex system, a microsphere assay mix is made with the intent that equal numbers of each bead classifier be present in the mix. In the above metaphor, we have the same number of each M&M color in the mix. In the Luminex system, classification of microspheres in the mix is by the ratio of intensity of 2 fluorophores bound into in the surface of the microspheres. In the above metaphor, classification is by color of each M&M candy. In the Luminex system, a sample of a microsphere mix is pipetted into wells in a multi-well plate containing sample. In the M&M metaphor, the well is represented by the swimming pool filled with a mix of M&Ms. In the Luminex system, the flow cytometer's acquisition probe is dipped into a well, sucking up a quantity of sample. In the M&M metaphor, the large barrel scooping up a sample of the M&M mixture out of the swimming pool corresponds to the probe sucking up sample. Some proportion of the Luminex instrument's acquired multiplexed microspheres from the sample makes it to the flow cell and then are counted after gating. In the M&M metaphor, this corresponds to the number of M&Ms that land inside the 6 foot diameter circle.

In the case of the Luminex flow cytometer, we know that the above "trial" will be repeated many thousands of times in the lifetime of an instrument. Consequently, outliers in the distribution occasionally will occur that prevent gathering of sufficient counts for a statistically valid sample. The distribution will be expected to show that larger numbers of microspheres per classifier in multiplexed mixtures require that larger numbers of microspheres be inserted into wells in order to raise the odds of acquiring a minimum number of microspheres in each sample. Those larger multiples of the acquisition counts rise non-linearly with the minimum acquisition counts required and with the number of elements in a multiplexed assay versus the number of microparticles put into each well. Another way of thinking of this study is, "If each color of M&M has a different assay on it, how many of each color are needed in the well to get a desired minimum count?"

This study was conceived to attempt to apply the results of the previous study by Jacobsen et al. [1] showing that larger numbers of microspheres for a classifier could increase the accuracy of results. The present study used realistic conditions likely to be found in a working Luminex lab, primarily, a 7-plex multiplexed assay and real serum sample. The hypothesis for this experiment was that it *would *be practical to obtain 1,000 microspheres for each of the 7 microsphere classifiers in the assay for all of the sample wells. The initial conception of this experiment was that it would be preliminary, a simple confirmation of the adequacy of an earlier preliminary experiment that showed a 5–10× multiple per classifier would work. However, significant problems became apparent. This study focuses exclusively on the ability of the Luminex system to obtain the desired counts of microspheres in a multiplex assay, and does not attempt to present data on relative confidence intervals or standard error obtainable using larger than normal numbers of microspheres per classifier in a multiplex assay.

## Methods

Preliminary trials were conducted (data not shown) using Luminex' (Luminex; Austin, TX) flow cytometer with carboxylate xMap™ microspheres (also Luminex) identifiers on MultiScreen HTS, BV 96 well plates (Millipore;Bedford, MA). Background on the Luminex system is available in the literature [2-9]. Varying identical counts were injected into a mix for each well to determine how many microspheres per classifier needed to be present per well. Results from these preliminary trials showed that when 1 or 2 microsphere classifiers were present at 5,000 or 10,000 microspheres per classifier per well, this was adequate to allow 1,000 microspheres of each classifier to be read. Consequently, for all further tests in this study, 10,000 microspheres per classifier per well were injected into the bead mix, with approximately 10% excess.

The multiplex assay that was used in the present study consisted of 7 different microsphere classifiers. Fluorescent intensity readings were not relevant to how many counts were obtainable and are not presented.

To determine the concentration of beads for each component bead classifier set, each bead classifier assay was vortexed in a 1 ml tube 10 μl was removed and added into 100 μl of PBS-Tween in a multi-well plate. A different well was used for each bead set. A Bio-Plex instrument (Bio-Rad: Hercules, CA) was used to count the number of beads acquired for a standard acquisition time and this figure was used to calculate bead concentration. (Bio-Rad is an OEM for the Luminex system.)

Highly characterized serum from a single Rhesus macaque with a Bio-Plex flow cytometer (Bio-Rad: Hercules, CA.) was utilized for this counting study. A 7-plex multiplex assay was used over 17 replicate wells with the Rhesus macaque serum. Into each of 17 replicate wells were injected 10,000 microspheres for each of the 7 assays making up the 7-plex multiplex. Acquisition attempted 1,000 microspheres per classifier per well for 3 minutes.

## Results

**Figure 1:**
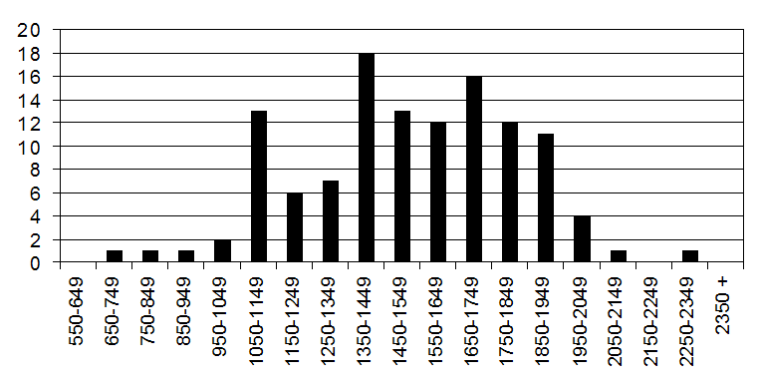
Microsphere counts distribution histogram for 7-plex replicate plate showing acquisition counts distribution for 17 wells with 7 assays per well. Acquisition target was set at 1,000 per microsphere classifier per well. X axis represents ranges of counts (bins). Y axis represents the number of assay (well) results having counts within the x axis bin range. Mean count for set = 1521. Standard deviation = 299. N = 119 (7 assays × 17 wells).

**Figure 2:**
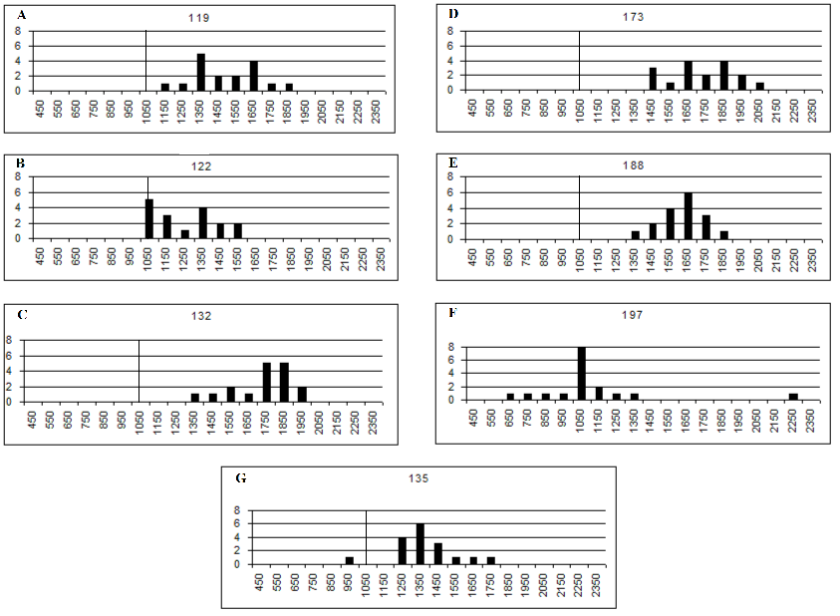
A-G: Microsphere counts distribution histograms for each classifier in the 7-plex replicate plate for 17 wells. Acquisition target was set at 1,000 per microsphere classifier per well. Vertical line is the 1000 value. X axis represents ranges of counts (bins), with only the start of each range shown. Y axis is number of assay (well) results having counts within the x axis bin range. For each graph N = 17 (1 assay × 17 wells). Note distribution for each classifier set, and outliers.

Distributions of counts per classifier for all wells and classifiers are shown as a collated distribution in Figure 1. Significant variation was found from well to well in the number of microspheres recoverable for individual assays, with assays not meeting the 1,000 count minimum. Individual classifiers set distributions are shown in Figure 2, Quite a few classifiers in each well had microsphere counts that were considerably above the 1,000 count minimum as well. The overall mean number of microspheres acquired was 1521. The standard deviation for the overall dataset was 299 and the overall N = 119. Fluorescent reading results from these assays are not shown or discussed.

## Discussion

Attempting to read large numbers of beads reliably and trying to apply the results from Jacobson et al. [1] by increasing bead count in a realistic 7-plex assay resulted in significant problems. Variance in microsphere counts acquired per classifier is to be expected, since the sampling of the mix for a multiplex assay with equal numbers of beads per assay is expected to be a multinomial distribution per Equation 1.

$$\begin{array}{l} Np_{k}=\frac{n!}{X_{1}!X_{2}!X_{3}!...X_{k}!}(p_{1}^{x_{1}}p_{2}^{x_{2}}p_{3}^{x_{3}}...p_{k}^{x_{k}})\\ Where:(p_{1}^{x_{1}}p_{2}^{x_{2}}p_{3}^{x_{3}}...p_{k}^{x_{k}})=p^{n}\\ because:p_{1}=p_{2}=p_{3}=p_{k} \end{array}$$

A multinomial distribution is the general case of which the more familiar binomial distribution is the case for two possibilities. The binomial distribution describes the probability of events such as the results of a pair of tossing dice. There is only one way that either a total of 2 or 12 can occur when tossing 2 dice, but a total of 7 can be made in three ways from 2 dice. The number of ways each number can occur out of all ways is the probability of occurrence, assuming fair dice. A multinomial expands this to the case of *k *items.

In the above multinomial equation 1, each N_p*k *_is the probability that any selected set of counts (example: for each color from a bag of 3 colors of M&Ms) will occur. If you take out 10 from the bag in a scoop, and have an equal number of each color in the bag, the result of Equation 1 gives you the probability that each specific outcome will occur. So if *n *= 10, there are three colors, and X_1 _= 1, X_2 _= 2, then X_3 _= 7, then *p*_1 _= (1/3) and so do all the other probabilities. So $p_{1}^{X_{1}}={\left(1/3\right)}^{1},p_{1}^{X_{2}}={\left(1/3\right)}^{2}$ and $p_{1}^{X_{3}}={\left(1/3\right)}^{7}$. Do this for each possible combination of X1, X2 and X3 and you have the theoretical probability distribution. Increasing the numbers in the above example of the bag of M&Ms to the numbers of microspheres in suspended microarray assays (or the swimming pool of M&Ms example) will give a theoretical distribution for the suspended microarray multiplexed assays. The normal (Gaussian) distribution will be expected to fit the distribution of counts for multinomials of the scale used in this study given a sufficient number of trials.

The results from these experiments indicated that there is a wide distribution in the number of spheres that will be obtained from different individual wells and for different microsphere classifiers within one well. A possible contributor to this problem could be that certain microsphere sets aggregated into dimers, trimers or larger aggregates, which would cause them to be gated out by the Bio-Plex instrument. No specific evidence was seen that this was occurring; however, the possibility exists for multiplexes. Another possible confounder could be that certain microsphere sets bound preferentially to the well or to the filter at the bottom of the well. Both of these possible confounders are reasons to believe that large counts may be problematic.

Some spread in variation is expected because of the imprecision in measuring concentration of microspheres of each component of the 7 classifier bead mix. This may be visible in the distributions of Figure 2, but, the presence of outliers (Fig 2F, 2G) for some classifiers suggests it may not be in all cases. This suggests the probable impracticality at present of measuring counts of all the components precisely enough to preclude significant variation due to a degree of unequal proportion of one classifier versus another in a multiplex. Thus, this factor would be expected to widen the distribution from the optimal potentially obtainable for a perfectly equal count multinomial distribution. It suggests that research into methods for standardizing counts of microspheres for each classifier of an assay in a multiplex might be worthwhile since it would be expected that a tighter distribution may allow fewer microspheres to be used per assay.

The Bio-Plex and Luminex systems have usually been used for microsphere counts in the range of 30 to 100 microspheres per classifier. (This corresponds to X_*k *_in Equation 1 for a multiplexed assay.) Typically, that means that 1000 to 2000 microspheres for each assay are put into each well to ensure reliably acceptable microsphere counts per sample. Occasionally, Bio-Plex and Luminex users see that there is difficulty collecting enough microspheres for one of the assays in a multiplex. Usually, this is attributed to not estimating correctly how many microspheres went into the bead mix. This may be correct, when most wells have a similar low count for one classifier of a multiplex. However, when low counts appear, whether it is a stochastic effect predominating, or something else may not be easily determined. Luminex's recommendations for each classifier are to the high side of 1000–2000, as stated above, but users generally get acceptable counts with lower quantities of microspheres per classifier and, in many instances, they try to conserve their beads. Probably, this acceptable user experience in conserving their assay beads is correlated with a lower order of multiplexing, as higher orders of multiplexing will be expected to result in a wider spread of counts.

In terms of considering the effect of this counts issue on diagnostics, when numbers of microspheres were acquired on the order of 1,000 or more, the MFI results were a bit more accurate, as shown by Jacobson et al. [1] (data not shown, refer to Jacobson et al. for well presented detail). However, in practice, acquiring 1,000 is complicated by microsphere count acquisition distribution. It can be argued that microsphere classifier sets for assays that did not make the cutoff value of 1,000 could still be used in some situations since the sample size is statistically valid and still large, with the caveat that the confidence in the result is just not quite as good. However, one assumes that the purpose of having higher precision/confidence results is to obtain more precise and reliable measurements for diagnostic purposes or in a clinical study or scientific experiment. Consequently, one needs to assume that protocols are expected to be strictly interpreted. So to make use of larger numbers of microspheres, one would need to loosen the protocol. Also, in studying carryover, results showed that random large carryover was a problem that would cause false positive and false negative tests [2] and acquiring more microspheres per classifier has no impact on that diagnostic problem.

Additionally, as a practical matter, high microsphere counts in multiplexes dictates that the numbers of microspheres for each classifier be so high in each well as to seriously impact cost, and result in much longer throughput times per plate because of the extra acquisition time required for each well (data not shown). Currently, these instruments do not quickly acquire 1000+ beads per classifier in a multiplex of significant size. Further, when the number of classifiers in the multiplex is increased to a high enough level, users can probably expect to see this stochastic problem more routinely when acquiring 30–100 counts per classifier using 1000–2000 beads per classifier in each well.

One Luminex instrument, the new FlexMAP 3D™ (not available for this study) can differentiate up to 500 different microsphere classifiers. If one were to create a distribution graph for this 500 microsphere classifiers, the outliers should be farther to the low and high regions of the graph given the same number of microspheres per classifier being put into the multiplex assay bead mix. Other concerns have been discussed [2] such as larger numbers of microspheres being associated with larger random carryover. The intent of Luminex in increasing the number of classifiers available in the FlexMAP 3D is to allow the development of very highly multiplexed assays, while conserving the sample. This is a good direction to take; it helps intraplexing and improves overall utility, but it is important to evaluate this new 500 classifier Luminex technology in light of this fundamental stochastic issue.

## Conclusion

Significantly multiplexed assays are subject to stochastic count variance causing a distribution of counts per classifier that has multiple ramifications. This is exaggerated by variances in both actual proportions of assay microspheres in the multiplex and actual ability to retrieve them. Consequently, increasing the number of microspheres acquired per classifier in the sample does not appear to effectively address the issues of reliability or improved precision of these assays in most situations. Nor does the precision solution proposed by Jacobson et al address another problem with unpredictable large carryover between sample wells [2] that is probably the most important. However, intraplexing assays [3] can address the issue of counts per classifier variance and precision allowing multiplexed assays to work with relatively low values of *n *for each assay in the multiplex at a high level of confidence in the precision of the final result.
